# Ameliorative and Renoprotective Effect of Electrical Stimulation on Blood Sugar, Blood Urea Nitrogen (BUN), Creatinine Levels, and the Islets of Langerhans Weight in Diabetic Mice

**DOI:** 10.1155/2022/7922892

**Published:** 2022-11-24

**Authors:** Suhariningsih Suhariningsih, S. Glory, F. Khaleyla, H. N. Kusumawati, M. Septriana, Y. Susilo, A. K. Yaqubi, S. D. Astuti, A. Syahrom

**Affiliations:** ^1^Department of Physics, Faculty of Sciences and Technology, Universitas Airlangga, Surabaya 60115, Indonesia; ^2^Biophysics and Medical Physics Research Group, Faculty of Sciences and Technology, Universitas Airlangga, Surabaya 60115, Indonesia; ^3^Department of Biology, Faculty of Mathematics and Natural Science, Universitas Negeri Surabaya, Surabaya, Indonesia; ^4^Traditional Medicine Study Program, Faculty of Vocational Study, Airlangga University, Surabaya 60286, Indonesia; ^5^Faculty of Engineering, Universitas Dr Soetomo, Surabaya 60118, Indonesia; ^6^Faculty of Science and Technology, Universitas Airlangga, Surabaya 60115, Indonesia; ^7^Department of Applied Mechanics and Design, Faculty of Mechanical Engineering, Universiti Teknologi Malaysia, Johor Bahru 81310, Malaysia

## Abstract

Diabetes mellitus (DM) is a chronic metabolic disease or disorder characterized by high blood sugar levels as well as impaired carbohydrate, lipid, and protein metabolism due to insulin function insufficiency. Insulin deficiency can be caused by impaired or deficient insulin production by Langerhans beta cells in the pancreas or by a lack of responsiveness of the body's cells to insulin. This study aims to the effects of electrostimulation on the ameliorative (improves disease manifestations) or renoprotective (protects the kidneys) in a diabetic rat model using noninvasive (electrical stimulation with the magnetic and nonmagnetic electrode) and invasive (using needles) methods. This study used 25 female rats, with a normal control group (KN), a diabetes control group (KD), a needle treatment group (A), an electro-stimulator treatment group with a magnetic electrode (M), and an ES group with a nonmagnetic electrode (ES) (L). The electro-stimulator used AES-05 with a magnetic field strength of 90 mT at two acupoints, Pishu (BL20) and Shenshu (BL23). The treatment was administered 12 times in one month with a therapy time of 6.6 minutes per session. Body weight and blood sugar levels were compared before and after the treatment. After treatment, the diameter of the islets of Langerhans, as well as levels of creatinine and blood urea nitrogen (BUN), was measured. Furthermore, statistical analysis was performed (*α* = 0.05). The results of this study showed that electrical stimulation treatments with needle-invasive, noninvasive magnetic electrodes, and nonmagnetic electrodes significantly reduced diabetic rats' blood glucose levels before and after the treatment. The analysis of the diameter of the islets of Langerhans revealed a significant difference between the treatment groups. The analysis of creatinine levels revealed a significant difference between groups, but creatinine levels in the group with the magnetic electrode (0.58 ± 0.17 mg/dL) were not significantly different from the control group (0.58 ± 0.07 mg/dL). The BUN test results revealed a significant difference compared with the diabetic control group, but no significant difference with the magnetic electrode treatment group. *Conclusion*. Based on the results, the most effective therapy for diabetes is a noninvasive method with magnetic (M) electrodes.

## 1. Introduction

Diabetes mellitus (DM) is a chronic metabolic disease or disorder characterized by high blood sugar levels as well as impaired carbohydrate, lipid, and protein metabolism due to insulin function insufficiency. Insulin deficiency can be caused by impaired or deficient insulin production by Langerhans beta cells in the pancreas. Diabetes is the fourth leading cause of death in the world. The number of cases in adults reached 285 million in 2010 and is expected to rise further [1]. So far, treating diabetes has involved giving patients insulin injections and oral antidiabetic medications. These expensive treatments can also result in anorexia, headaches, nausea, and dizziness in some people [2, 3]. Acupuncture and electric needles have both been used in research for the invasive diagnosis and therapy of organ disease [4–6], and noninvasively using light [7–9]. The human body is equipped with an electrical system [10]. Each has an electric voltage from the brain to the heart with a negative charge in the cell membrane and a positive charge on the inside and outside of the cell membranes [11]. As a result, the human body can be described as a bioelectric system [12]. The presence of organ dysfunction has been detected using electricity in the body [4, 5]. An electro-stimulator is an electronic device that generates electrical stimulation with a specific frequency, amplitude, and waveform for use in therapy [13]. Electro-stimulator devices are useful as therapeutic tools for improving blood circulation, increasing muscle strength, relieving pain, and restoring damaged tissue [14]. The effectiveness of electro-stimulator therapy is determined by the intensity (voltage and current), frequency, and duration of stimulation [15, 16].

The accuracy of the physical parameters of the electro-stimulator is determined the success rate of therapy. Because of its small pulse width, the spike waveform is known to be an effective electro-stimulator waveform used in therapy for Parkinson's disease [17]. The therapeutic energy dose in an electro-stimulator is calculated by multiplying the stimulation power (*P*) by the therapy time (t). The electrical power determined by the square of the stimulation voltage (*V*_eff_) divided by the body's resistance between the two electrodes is the stimulatory power in electro-stimulator therapy [18, 19]. Acupuncture stimulation has been shown to affect rat blood sugar concentrations [20–22]. Another study found that electrical stimulation of acupuncture points can lower body weight while increasing blood glucose levels in obese people [23]. A wireless telemetric sensor system has also been used in the implementation of therapy and monitoring diabetic patients [24]. So far, DM has been treated with oral antidiabetic drugs such as sulfonylurea and thiazolidine, as well as insulin injections. e form of drugs and insulin injections, have several disadvantages, which can cause hypoglycaemia, weight gain, and gastrointestinal disorders [25]. Therefore, it is necessary to have other alternatives for the treatment of diabetes mellitus apart from drugs and insulin injection, one of which is to use electrical stimulation with electro-stimulator.

This study aims to determine the efficacy of electro-stimulators in a diabetic rat model for ameliorative (improves disease manifestations) and renoprotective (protects the kidneys) effects using noninvasive (magnetic and nonmagnetic) and invasive (needle) methods. The study's hypothesis is that , therapy using an electro-stimulator has a significant impact on blood sugar levels and the size of the Langerhans islets.

## 2. Materials and Methods

### 2.1. The Materials Samples

In this study, 25 adult female strain BALB/c mice were used. The samples were grouped into 2 groups, namely the healthy group (5 mice) and the diabetic group (20 mice). All experimental animals were acclimatized for seven days before treatment and food and drink were available at all times during the trial. The diabetic groups were induced orally with lard on a high-fat diet before being streptozotocin-induced diabetic mice (STZ). Along with the intraperitoneal dose of STZ, the rats were fed a commercial meal designed for rodents that were enhanced with animal fat (lard) and sugar (to boost the caloric value). A dose of STZ that was a little bit lower than normal was employed to create DM1. According to our hypothesis, the animals would exhibit the major signs of DM2 in people, such as hyperglycemia, insulin resistance, a changed lipid profile, and other hyperglycaemic effects (such as glycosuria, polyuria, and thirst). The lard dose was 0.3 mL/30 g BW every two days for 20 days, the mice were induced with STZ dissolved in citrate buffer of pH 4.5 intraperitoneally for 5 days at a dose of 30 mg/kg BW. The high-fat diet rat should experience consistent hyperglycemia for at least 130 days after receiving this dose of STZ. The STZ dosage is crucial because it results in a model that more closely resembles T1, and if it is too high, mortality increases. It is recommended by Chen et al. [26] to administer two smaller doses of STZ (30 mg/kg, i.p) at weekly intervals. 85% of the rats treated with this regimen develop diabetes with mean fasting blood glucose levels of 14 mmol/L (252 mg/dl). The ideal dose for 12-week-old Sprague-Dawley rats fed a high-fat diet for 8 weeks was 30 mg/kg STZ i.p. On the 7^th^ and 14^th^ days after induction, blood sugar levels in mice were measured. Diabetes was defined as a blood sugar level greater than 200 mg/dL in diabetic mice. The blood glucose level was measured using a self-glucose monitoring kit (MEDISAFE, TERUMO).

### 2.2. Instrument

**Figure 1:**
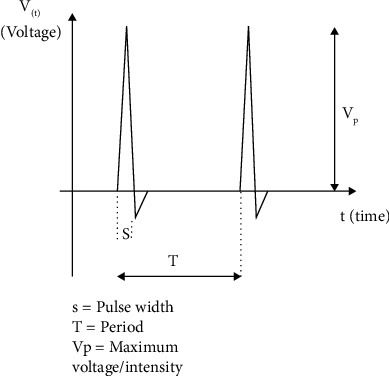
Spike pulse of the electro-stimulator.

An electro-stimulator is a tool used in modern acupuncture therapy that uses electric current pulses to stimulate acupuncture points. The electro-stimulator used is AES-05 which has a spike pulse output with a pulse width of 0.2 ms, a peak voltage of 0–300 V, and an output frequency of 90 Hz. The electrode used is a permanent magnet with a diameter of 20 mm. AES-05 positive electrode was placed at the BL20 acupuncture point and the negative electrode was placed at the BL23 acupuncture point which was carried out on the left and right sides of the rat's body [21]. A frequency of 6 Hz can stimulate growth due to the low frequency of stimulation [18].  Figure 1 shows the spike pulse of the electro-stimulator (AES-05).

Energy dose

(1) $$\begin{array}{c} D\left(Joule\right)=P\left(Watt\right)\times t\left(second\right);Watt=\frac{Joule}{second}\text{.} \end{array}$$

Power

(2) $$\begin{array}{c} P\left(watt\right)=I_{eff}\times V_{eff}\;or\;P=\frac{{\left(V_{eff}\right)}^{2}}{R}\text{.} \end{array}$$

Effective voltage for low frequency (Puk)

(3) $$\begin{array}{c} V_{eff}=V_{p}\sqrt{\frac{s}{2T}\;}\text{.} \end{array}$$

From equation (1),

(4) $$\begin{array}{c} the\;therapy\;time\;t=\frac{D}{P}second\text{.} \end{array}$$

So that the therapeutic time in this study is

(5) $$\begin{array}{c} t=\frac{D\;R}{\left(1/2\right){\;V}_{p}^{2}\;Sf}\text{.} \end{array}$$

According to equation (5) in this study if each sample therapy received a dose of 1 joule, then the time required for therapy was 6.6 minutes.

### 2.3. Experimental Protocol

**Figure 2:**
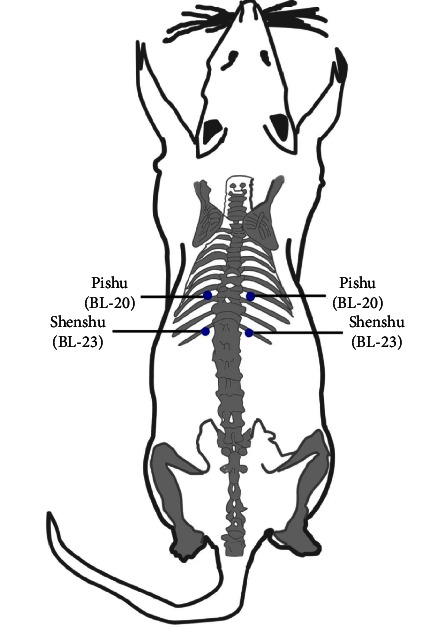
Location of acupuncture points (Pishu, Shenshu) on the backs of mice [20].

**Figure 3:**
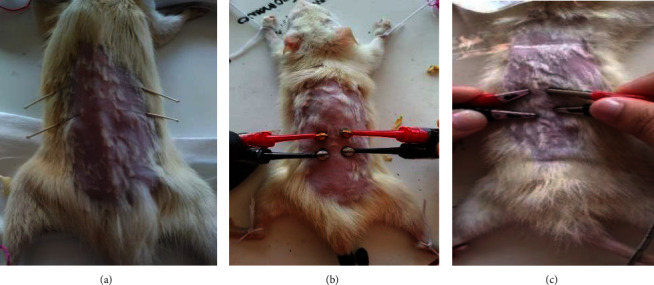
(a) Electro stimulator therapy with needle therapy (A), (b) magnetic electrodes (M), and (c) nonmagnetic electrodes (L).

Normal control (KN), diabetes control (KD), needle therapy (A), electro-stimulator therapy with magnetic electrodes (M), and electro-stimulator therapy with nonmagnetic electrodes (L) were the five groups of experimental animals. Each treatment group had five mice. The mice's weight and blood sugar levels were measured before the electro-stimulator treatment. Electro-stimulator treatment was given to mice at the acupuncture points Shensu (BL23), the Shu point of the kidney, Pishu (BL20), and the Shu point of the spleen organ (Figure 2) [20]. Group M received electro-stimulator therapy with magnetic electrodes attached to the Pishu and Shensu acupuncture points (Figure 3(a)), group L received electro-stimulator therapy with nonmagnetic electrodes attached to the Pishu and Shensu acupuncture points (Figure 3(b)), and group A received acupuncture needle treatment without electrical stimulation at the Pishu and Shensu points (Figure 3(c)). The experimental animals were treated 12 times over a month. If therapy is given 1 joule of energy each time (6.6 minutes), the total energy transferred into the body over a month is 12 joules.

### 2.4. Collection of Samples

The experimental animals' body weight was measured again after treatment. Random intracardiac blood samples were tested for blood urea nitrogen (BUN), blood glucose, and creatinine. The rat B-cell leukemia/lymphoma 2 bioassay technology laboratory enzyme-linked immunosorbent assay (ELISA) kit was used to measure the quantity of Bcl-2 (BTL, China). First, we figured out how many pre-coated ELISA plates were there. Microwells were only ever used once before being thrown away. Next was preparing the reagents for the standard solution, including the standard diluent, washing buffer concentrate, streptavidin-HRP, substrate solution A, substrate solution B, stop 61 solutions, and biotinylated rat Bcl-2 antibody. The standard solution was then diluted with the standard diluent in accordance with the ELISA kit's protocol. Because the standard solution already has an antibody that has been biotinylated, no additional antibody was given; 50 l of the standard solution was supplied to the standard well instead. Then, 10 l of anti-Bcl-2 antibody was added to the sample sump and 50 l of streptavidin-HRP to the sample wells and standard wells after placing 40 l of serum sample in each well in accordance with the marks on each well. A plastic cover was placed over the plate, and it was then incubated at 37°C for 60 minutes. The plate was emptied and washed five times with washing buffer. Then, 50 l of substrate solution A was added to each well, and 50 l of substrate solution B was added to each well and then incubated for 10 minutes at 37°C in a dark room. A positive response showed a blue color. The reaction was stopped by adding 50 l of stop solution to each well, the blue color immediately turned yellow. The reading of the OD (optical density) value was carried out with an ELISA reader at a wavelength of 450 nm. Then, a standard curve was made to get the *R*-value and the equation that was used to measure the concentration of Bcl-2. To measure the diameter of the islets of Langerhans, the pancreas was fixed and processed into histological preparations with haematoxylin-eosin staining [10].

### 2.5. Histology of Islets of Langerhans

**Figure 4:**
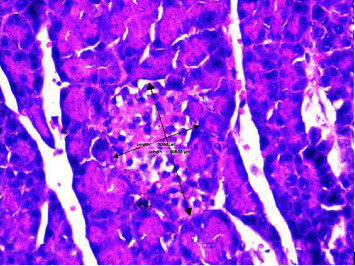
Method for measuring the diameter of the islets of Langerhans in the pancreas.

The diameter of the Langerhans islets was measured on serial pancreatic histological sections. The diameter of Langerhans islets used the largest size, which is then incorporated into the calculation results of subsequent data. The number of Langerhans islands was determined by magnifying the entire field of view 100 times. At 400x magnification, the diameter of the Langerhans discharge and the number of beta cells were counted on 5 Langerhans islets. An ordinary Nikon E100 light microscope with a 12-megapixel Opti lab Advance Plus digital camera and Image Raster 3.0 image processing software is used for this examination.  Figure 4 depicts how to measure the diameter (length and width) of the pancreatic islet of Langerhans. Placement of the measuring line perpendicular to the islet of Langerhans' long axis. (400x magnification; haematoxylin and eosin stain).

### 2.6. Data Analysis

Data on blood sugar, creatinine, and BUN levels were statistically analysed using the analysis of variance (ANOVA) followed by the Tukey posthoc test with *α* = 0.05.

## 3. Results

### 3.1. Effects of Electro Stimulus Therapy on Random Sugar Levels and Pancreatic Islet Diameter

**Figure 5:**
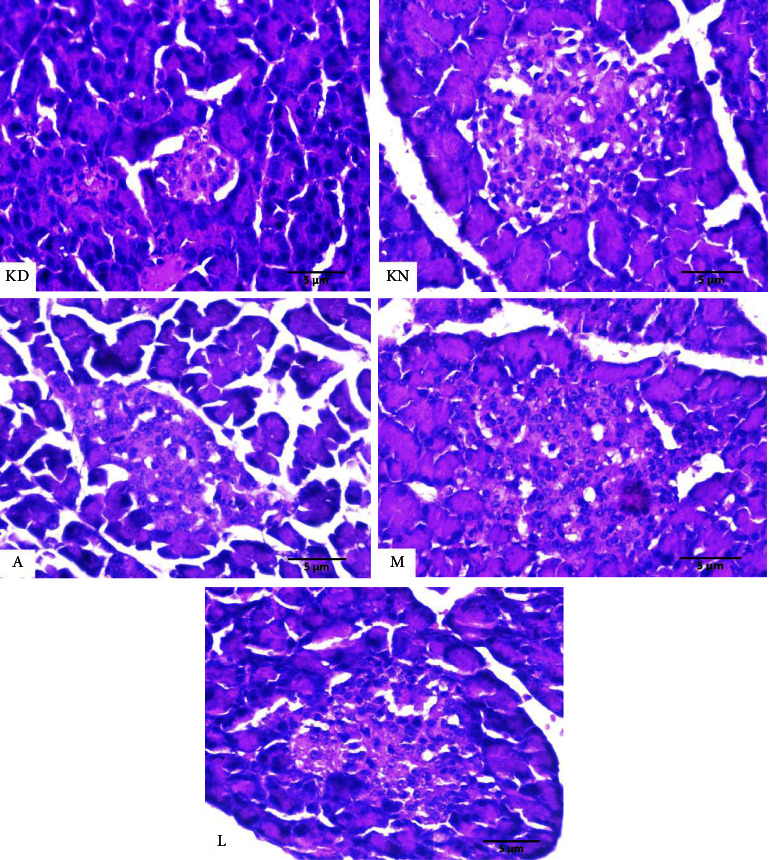
Comparison of the diameters of the islets of Langerhans from all treatment groups normal control (KN), diabetes control (KD), needle therapy (A), electro-stimulator therapy with magnetic electrodes (M), and electro-stimulator therapy with nonmagnetic electrodes (L). The magnification of the image is 400x.

Table 1 shows the effect of electro-stimulus treatment on the random sugar levels of mice and the diameter of the islets of Langerhans. The random blood sugar levels of the diabetic mice in the KD, A, M, and L groups were all over the cutoff (>200 mg/dL) at the beginning of treatment, despite statistical differences between groups. Blood sugar levels in all after needle acupuncture and electro-stimulator treatment, treatment groups returned to normal. The *T*-test results on blood sugar level data revealed a significant difference in the treatment groups before and after A, M, and L therapy but not in the KD and KN groups. The ANOVA test results on blood sugar levels after treatment revealed no significant difference from normal controls (KN). In this study, there was no placebo group. In Table 1 control group without treatment is used as a comparison for the treatment group, whether the treatment was successful. The control group was measured at the same time as the treatment group.  Figure 5 compares the diameters of the Langerhans islets in the KN, KD, A, M, and L treatment groups.

### 3.2. Renoprotective Effects of Electro Stimulus on BUN and Creatinine

Table 2 shows the results of measuring blood urea nitrogen (BUN) and creatinine levels. BUN levels in the treatment groups A, M, and L were not significantly different from the normal control group, according to statistical analysis (KN). The L group did not differ significantly from the KN and KD groups. The creatinine level data analysis revealed that the treatment groups A, M, and L did not differ significantly from the negative control (KN). The creatinine level in the KD group was significantly higher than in the KN group. Creatinine levels in group L did not differ significantly from those in KN and KD.

## 4. Discussion

Indonesian Endocrinology Society (Perkeni) defines diabetes mellitus as having random glucose levels above 200 mg/dL, whereas normal randomized glucose levels are 70–139 mg/dL 2 hours after eating [27]. Diabetes was present in all STZ-induced mice, as evidenced by random blood sugar levels above 200 mg/dL in KD [10] and all treatment groups. According to the findings (Table 1), the three types of therapy were effective in lowering random blood sugar levels in STZ-induced type I diabetes mellitus mice.

Acupuncture has been shown in several studies to reduce glucose levels in patients with diabetes mellitus. In a previous study, diabetic mice who received electroacupuncture therapy at the PC6 + ST36 point had significantly lower blood sugar levels than those who did not receive treatment. Electroacupuncture is thought to improve glycaemic control in a diabetic animal model by inducing IGF1R pathway upregulation [28]. Acupuncture may also help control lipid metabolism in mice with nonalcoholic fatty liver disease (NAFLD), a condition linked to the development of diabetes mellitus. Acupuncture at points ST36, KI1, and CV4 has been shown to reduce lipid absorption in the intestines of obese mice [29].

In diabetic mice, the three therapies decreased blood glucose levels and increased islets of Langerhans size. Among the three treatments for diabetic conditions, electro-stimulator therapy with magnetic (M) electrodes is the most effective. In this study, creatinine and BUN levels were also measured as indicators of the treatment's renoprotective ability. Creatinine is a by-product of muscle protein and an end-product of muscle metabolism that is excreted in the urine at the same rate as it is released from the muscles. The kidneys excrete creatinine through a combination of filtration and secretion. Creatinine levels that are higher than normal indicate poor kidney function [30]. Induction of diabetes mellitus using streptozotocin causes organ damage due to increased levels of reactive oxygen species, which can be indicated by changes in BUN and serum creatinine levels [31].

Due to the fact that women normally have higher muscle mass than men, prior studies also revealed that women's creatinine levels were lower than men's. In the present study, low creatinine levels in the KD group could reflect a decrease in kidney muscle mass caused by aging or liver issues. Because they are small and go through a menopausal phase, female rats were used in this investigation [32]. Treatments M and L were the only two that could return creatinine levels to normal. Despite being significantly different from the control group, the L group's creatinine levels remained within the normal creatinine threshold for mice [33]. The three treatments, on the other hand, had no significant effect on BUN levels in diabetic mice.

Based on the other study, stimulation of the acupuncture point, Pishu (BL 20) using a 650 nm and 980 nm laser significantly reduced blood sugar levels in diabetic Wistar rats [34]. In traditional Chinese medicine, the Pishu point (BL20) is used to awaken, while the Shenshu point (BL23) is used to awaken the kidneys. Both organs are associated with the pancreas which controls insulin production and the development of diabetes mellitus. Traditional acupuncture considers suppressing ROS production by regulating the PKC/P66shc signalling pathway in diabetic rats, whereas electroacupuncture has previously been shown to reduce ROS by suppressing nitric oxide production and thereby decreasing NADPH oxidase activity [35]. This regulation, which affects creatinine and BUN levels, can reduce pancreatic organ damage [36].

## 5. Conclusions

Electrical stimulation treatments with needle invasive, noninvasive magnetic electrodes, and nonmagnetic electrodes significantly reduced blood glucose levels in diabetic rats before and after treatment. The diameter of the Langerhans islets revealed a significant difference between the treatment groups. The analysis of creatinine levels revealed a significant difference between groups, but creatinine levels in the magnetic electrode group did not differ significantly from the control group. The BUN test results showed a significant difference when compared to the diabetic control group, but no difference was seen when compared to the magnetic electrode treatment group.

## Figures and Tables

**Table 1:** Effects of electrostimulation therapy on random blood sugar levels and diameter of the islet of Langerhans in diabetic mice.

No.	Treatment group	Random blood sugar level (mg/dL)^*∗*^		Diameter of the islet of Langerhans (*µ*m)^*∗*^
		Before treatment	After treatment	
1	Normal control (KN)	107.80 ± 5.36^a^	119.20 ± 7.98^a^	112.25 ± 19.87^ab^
2	Diabetes control (KD)	313.80 ± 59.76^b^	336.60 ± 65.60^b^	90.78 ± 21.35^b^
3	Needle therapy (A)	448.00 ± 32.91^c^	124.80 ± 5.36^a^	108.74 ± 20.45^ab^
4	Magnetic electrodes (M)	489.00 ± 73.55^c^	112.20 ± 19.52^a^	133.26 ± 23.07^a^
5	Nonmagnetic electrodes (L)	450.80 ± 49.37^c^	101.20 ± 4.15^a^	103.17 ± 18.85^ab^

**Table 2:** Renoprotection effect electrical stimulation therapy for blood urea nitrogen (BUN) and creatinine levels.

No.	Treatment group	Blood urea nitrogen (mg/dL)	Creatinine level (mg/dL)
1	Normal control (KN)	37.70 ± 1.90^ab^	0.58 ± 0.07^bc^
2	Diabetes control (KD)	40.60 ± 20.34^a^	0.24 ± 0.21^a^
3	Needle therapy (A)	40.30 ± 0.88^b^	0.75 ± 0.09^c^
4	Magnetic electrodes (M)	40.98 ± 3.33^b^	0.58 ± 0.17^bc^
5	Nonmagnetic electrodes (L)	38.10 ± 0.48^ab^	0.45 ± 0.03^ab^

## Data Availability

https://drive.google.com/drive/folders/1uDcOxAnY_nVJNX5Iu4i40QvMwWgPPnU8?usp=sharing.
